# Supplementary material for: Terminomics Methodologies and the Completeness of Reductive Dimethylation: A Meta-Analysis of Publicly Available Datasets
Source: Proteomes. 2019 Mar 29;7(2):11. doi: 10.3390/proteomes7020011 (PMC6631386; doi:10.3390/proteomes7020011)
Supplement: Supplementary file 1 [file proteomes-07-00011-s001.pdf]

Supplementary table one: The PRIDE datasets used for reanalysis and the relevant parameters used in the search. Variable modifications and mass tolerences were the same as those used in the original publication.

Data set reference	Search engine and release version	Name of sequence database and release version	# of entries in the database searched	Specificity of all proteases used to generate peptides	# of missed and/or non-specific cleavages permitted	List of all fixed modifications	List of all variable modifications	Mass tolerance for precursor ions (ppm)	Mass tolerance for fragments ions (Da)
PXD002785<br>PXD003833<br>Biniossek et al.	PeaksDB v8.5	UniProt *E. coli*	4448	Trypsin, semi specific	5	None	Dimethylation<br>Carbamidomethylation<br>Dimethyl-$C^{13}HD^{2}$<br>Heavy formaldehyde + cyanobordeuteride	10	0.05
PRD000055<br>Boersema et al.	PeaksDB v8.5	UniProt Rat and Mouse	Rat: 30173<br>Mouse: 51608	Trypsin, semi specific	5	None	Oxidation (M)<br>Dimethylation<br>Carbamidomethylation<br>Heavy dimethyl (triplex)<br>Intermediate dimethyl (triplex)	20	0.1
PXD005920<br>Roperto et al.	PeaksDB v8.5	UniProt *M. bovis*	10436	Trypsin, semi specific	5	None	Oxidation (M)<br>Dimethylation<br>Carbamidomethylation<br>Heavy dimethyl (triplex)<br>Intermediate dimethyl (triplex)	15	0.05
PXD003298<br>Salih et al.	PeaksDB v8.5	UniProt Human	70266	Trypsin, semi specific	5	None	Oxidation (M)<br>Dimethylation<br>Carbamidomethylation<br>Intermediate dimethyl (triplex)	15	0.05
PXD004654<br>Varano et al.	PeaksDB v8.5	UniProt *O. anthropi*	27935	Trypsin, semi specific	5	None	Oxidation (M)<br>Dimethylation<br>Carbamidomethylation<br>Intermediate dimethyl (triplex)	15	0.05
